# LuQi Formula Ameliorates Myocardial Fibrosis by Suppressing TLR4/MyD88/NF-*κ*B Pathway and NLRP3 Inflammasome Activation in Mice with Myocardial Infarction

**DOI:** 10.1155/2022/5867987

**Published:** 2022-03-11

**Authors:** Xiaoqing Zhang, Huiyan Qu, Tao Yang, Qian Liu, Dandan Zhao, Wenrui Liu, Tian wang, Hua Zhou

**Affiliations:** ^1^Institute of Cardiovascular Disease of Integrated Traditional Chinese and Western Medicine, Shuguang Hospital Affiliated to Shanghai University of Traditional Chinese Medicine, Shanghai, China; ^2^Department of Cardiovascular Disease, ShuGuang Hospital Affiliated to Shanghai University of Traditional Chinese Medicine, Shanghai, China

## Abstract

**Background:**

Myocardial fibrosis caused by myocardial infarction (MI) is the key factor leading to cardiac remodeling; nod-like receptor family pyrin domain-containing 3 (NLRP3) plays an important role in regulation of myocardial injury; however, its relationship with TLR4/MyD88/NF-*κ*B signaling pathway is largely unreported. In recent years, traditional Chinese medicine (TCM) prevention and treatment of cardiovascular diseases has shown its unique advantages and broad application prospects. LuQi Formula (LQF) has been used for more than 20 years in Shuguang Hospital (Shanghai, China), and it was confirmed that it can improve the clinical symptoms of patients after MI. Here, we investigated the mechanism of LQF by suppressing NLRP3 inflammasome activation and TLR4/MyD88/NF-*κ*B pathway in mice with MI.

**Purpose:**

The purpose of this study was to verify the positive effects of the LQF in ameliorating myocardial fibrosis and inflammasome infiltration in the MI mice in vivo.

**Methods:**

Forty mice were randomized into four groups: the sham group, the MI group, the LQF group, and the perindopril group (*n* = 10 per group). Left anterior descending (LAD) coronary artery ligation was performed in all groups except the sham group. The mice were treated with LQF after MI. After 4 weeks, LDH, cTnI, IL-1*β*, and IL-18 were measured by enzyme-linked immunosorbent assay (ELISA) kit, and cardiac function was evaluated by echocardiography. Hematoxylin and eosin (H&E) and Masson staining were used to evaluate the myocardial injury and fibrosis. Western blot was used to evaluate the expression of collagen I, *α*-SMA, NLRP3 inflammasome, and TLR4/MyD88/NF-*κ*B signaling pathway. Immunohistochemical analysis was used to further detect the expression of Fibronectin, *α*-SMA, collagen I, collagen III, NLRP3, and NF-*κ*B in myocardial tissue.

**Results:**

Compared with the MI group, the ejection fraction (EF) and fractional shortening (FS) in the LQF group were significantly improved, while the left ventricular end diastolic diameter (LVEDd) and left ventricular internal dimension systole (LVIDs) were significantly decreased. The representative staining of H&E and Masson showed that treatment with LQF could effectively reduce myocardial injury and fibrosis. ELISA results showed that serum LDH, cTnI, TNF-*α*, IL-18, and IL-1*β* in LQF group were significantly lower than those in MI group. The western blot results showed that the expressions of collagen I and *α*-SMA were decreased significantly in the LQF group. Moreover, the expressions of NLRP3 inflammasome and TLR4/MyD88/NF-*κ*B signaling pathway were downregulated in the LQF treatment group.

**Conclusion:**

Our results suggested that LQF could significantly improve cardiac function and ameliorate myocardial fibrosis. In addition, we found that LQF could downregulate the TLR4/MyD88/NF-*κ*B signaling pathway and then inhibit the activation of NLRP3 inflammasome, suggesting that LQF alleviated cardiac fibrosis by decreasing the TLR4/MyD88/NF-*κ*B signaling pathway and then inhibited NLRP3 inflammasome activation in MI mice, which indicates potential therapeutic effect of LQF on patients with MI.

## 1. Introduction

MI is one of the most common causes of death across the globe; percutaneous coronary intervention therapy can effectively reduce the mortality of patients with MI and hence becomes the most important treatment strategy for MI. However, the patients with MI still have an increased incidence of heart failure (HF) [1, 2]. The cardiomyocyte is nonrenewable [3]; however, there is still a lack of specific drugs for the treatment of myocardial tissue injury after MI. Analyzing the mechanism of myocardial injury after MI and looking for new therapeutic drugs and key therapeutic targets to guide clinical treatment have attracted extensive attention of researchers.

A large number of studies have shown that inflammatory response is an important cause of myocardial injury, which is across the whole process of MI [4, 5]. NLRP3 inflammasome is an important part of the innate immune system. It is a macromolecular protein complex composed of NLRP3, apoptosis-associated speck-like protein containing a caspase recruitment domain (ASC), and effector protein cysteine aspartate protease-1 precursor (pro-caspase 1). NLRP3 mediates caspase-1 activation after inflammasome activation and then promotes interleukin-1*β* (IL-1*β*) and interleukin-18 (IL-18) maturation and secretion, as well as simultaneous lysis of gasdermin D (GSDMD) induced cell pyroptosis [6]. The classical NLRP3 inflammasome activation pathway includes two stages: initiation and activation. In the initiation stage, toll-like receptor 4 (TLR4) mainly recognizes extracellular pathogen associated molecular patterns (PAMPs) or danger associated molecular patterns (DAMPs), and nuclear factor kappa B (NF-*κ*B) is activated by the downstream signaling molecule myeloid differentiation factor 88 (MyD88), finally inducing the expression of NLRP3 and IL-1*β* precursors. The activation phase is characterized by multiple stimuli that promote assembly and activation of the NLRP3 inflammasome [7]. Thus, TLR4/MyD88/NF-*κ*B pathway is crucial for NLRP3 inflammasome activation. In recent years, a large number of studies have shown that TLR4/MyD88/NF-*κ*B pathway and NLRP3 inflammasome mediate the occurrence of inflammatory response in MI, resulting in myocardial injury [8]. Therefore, inhibition of the TLR4/MyD88/NF-*κ*B signaling pathway and NLRP3 inflammasome can reduce the inflammatory response and improve ischemia/reperfusion injury [9, 10].

LQF is a traditional Chinese medicinal formula to treat myocardial fibrosis (Chinese national patent no. ZL2014102933164), and it has been used for 20 years in the clinic. Previous experimental studies showed that LQF significantly improved cardiac function and downregulated the expression of NLRP3 inflammasome in the MI mice; however, the upstream mechanism of activating NLRP3 inflammasome is still unclear [11]. Angiotensin-converting enzyme (ACE) inhibitors are one of the main treatment options for patients with MI, which can effectively improve the cardiac function of patients with MI, so we chose perindopril as the positive control [12, 13]. In this study, we aimed to investigate the effects of LQF on myocardial fibrosis after MI and its underlying mechanisms. We demonstrated that LQF can efficiently inhibit TLR4/MyD88/NF-*κ*B signaling pathway activation and thereby inhibit NLRP3 inflammasome activation and alleviate myocardial fibrosis in MI mice.

## 2. Materials and Methods

### 2.1. Animals and Drugs

Male C57BL/6J mice (20 g–25 g) were obtained from experimental animal center of Shanghai University of TCM (Shanghai, China). Our experimental procedures were conducted in accordance with safe animal testing specifications (Safety Certificate Number: SYXK-HU-2020-0009; Animal Ethics Code: PZSHUTCM200807012). The mice were kept in a humanized environment with favorable temperature (23 ± 2°C), humidity (50 ± 5%), and 12 h/12 h light/dark cycle and fed with normal diet and water ad libitum. LQF is composed of antler, safflower, *Astragalus membranaceus*, *Codonopsis pilosula*, Cassia Twig, and Semen Lepidii. The raw herbs were purchased from Shanghai Kangqiao Chinese Medicine Tablet Co., Ltd. (Shanghai, China) and identified by Dr. Zhengtao Wang from Pharmacy Department, Shanghai University of TCM. LQF consists of nine traditional Chinese herbal medicines. The six herbs were dissolved in distilled water and concentrated to 1.78 g/kg. Perindopril tablets (4 mg/tablet) were purchased from Servier Pharmaceuticals Co., Ltd. (Tianjin, China) and used as a positive control. Perindopril was dissolved and diluted by saline with a concentration of 0.06 mg/mL of solution.

### 2.2. Mouse Model of MI and Grouping

Forty mice were fed adaptively for one week before modeling. First, the mice were anesthetized with 2% isoflurane (RWD Life Science Co., Guangdong, China) and fixed in supine position. Then, the mice were ventilated with a ventilator (Harvard Apparatus) and the thoracic cavity was opened (respiratory rate, 110/min). The LAD coronary artery was ligated with 7–0 surgical suture, and the thoracic cavity was closed and sutured. The mice in sham operation group were only threaded without ligation. After operation, whether the “J point” of ECG was raised was observed and the myocardium below the ligation site was obviously white, which can be used as a sign of successful model. After modeling, except the sham operation group, all the mice were randomly placed in each cage. A total of 40 mice were randomly divided into the sham group, the MI group, the LQF group, and the perindopril group. The mice in the sham group and the MI group were given normal saline (0.1 ml/10 g), and the mice in the LQF group (1.65 g/ml) and the perindopril group (0.06 mg/ml) were, respectively, administered orally. All mice underwent treatment for four weeks at the same time every day.

### 2.3. Ultrasound Echocardiography

Anesthetized mice were maintained by inhalation of 2.5% isoflurane. Echocardiography was detected by high-resolution small animal ultrasound echocardiography (Netherlands Philips IE33). The parasternal short axis section of the left ventricle and the left ventricular motion was recorded by M-mode ultrasound, LVIDs and LVEDd were measured, and EF and FS were calculated to evaluate cardiac function.

### 2.4. Western Blotting

Left ventricular tissues were homogenized in tissue grinder in lysis buffer supplemented with protease and phosphatase inhibitors, and the myocardial homogenate was prepared using tissue homogenizer. The homogenate was lysed in ice for 30 min and centrifuged at 8000×g and 4°C for 10 min, and the supernatant, namely, total myocardial protein, was collected. The protein concentration was determined by BCA (Thermo Fisher Scientific, Waltham, MA, USA; A53225) method, and the sample buffer was added and boiled at 95°C for 10 min. The protein bands were transferred to PVDF membrane after SDS-PAGE gel electrophoresis. The protein bands were blocked with 5% skim milk powder at room temperature for 1 h and then incubated overnight with primary antibody at 4°C. On the next day, they were incubated with different secondary antibodies. The protein images were detected by ECL method after washing the membranes (Tanon Science and Technology Co., Shanghai, China). In the present study, the primary antibodies included collagen I (1 : 1000, Abcam, ab270993), *α*-SMA(1 : 1000, Abcam, ab5694), TLR4 (1 : 200, ABclonal, A17436), MyD88 (1 : 1000, Affinity, AF5195), P-NF-KB (1 : 1000, CST, 3033T), ASC (1 : 1000, CST, 67824S), NLRP3 (1 : 1000, CST, 15101S), and caspase-1 (1 : 1000, Abcam, ab179515). GAPDH (1 : 20000, Bioworld, AP0066) was used as loading control, and ImageJ (NIH, Bethesda, MD, USA) software was used to analyze the relative expression of target protein.

### 2.5. Histological Staining

The myocardial tissue of mice fixed with formaldehyde was embedded in paraffin and cut into 5 *μ*m sections. H&E and Masson staining (Beyotime, Shanghai, China) were performed as previously reported [11]. After sealing with neutral gum, the myocardial pathological changes were observed under the microscope (Pannoramic 250/MIDI, 3DHISTECH, Budapest, Hungary).

### 2.6. Immunohistochemical (IHC) Analysis

Paraffin sections were deparaffinized, and then tissue sections were put in sodium citrate antigen repair buffer for high-temperature antigen repair. After cooling, they were washed in PBS for 3 times and then put in 3% hydrogen peroxide solution to block endogenous peroxidase with 5% BSA, followed by adding the prepared primary antibody, overnight incubation at 4°C, adding the secondary antibody, and then washing with PBS for 3 times, adding the diaminobenzidine (DAB), and finally using hematoxylin counterstaining and neutral gum sealing. Images were observed and collected under a microscope (Pannoramic 250/MIDI, 3DHISTECH, Budapest, Hungary).

### 2.7. ELISA

Blood collected from mice was incubated in room temperature for 30 min and then was centrifuged at 2500xg for 15 minutes. The concentrations of LDH (SG-30229, Sinogene, China), cTnI (H149-2, Nanjing Jiancheng Bioengineering Institute, China), TNF-*α* (70-EK282/4-96, Multisciences, China), IL-1*β* (70-EK201B/3-96, Multisciences, China), and IL-18 (70-EK218-96, Multisciences, China) were quantified with the indicated kits according to the manufacturer's instructions. In brief, all reagents and samples were restored to room temperature. 100 *μ*l HRP-conjugated secondary antibodies were added to 50 *μ*l of the standard substance or protein samples in the wells of the plates and then incubated at room temperature for 1 h. After that, the wells were washed with 300 *μ*l washing buffer for six times and incubated with the 100 *μ*l TMB solution in dark for 30 min. Finally, the optical density (OD) value was quantified under 450 nm within 30 min.

### 2.8. Statistical Analysis

All data are expressed as the mean ± standard deviation (SD); statistical analyses between two groups were carried out using Student's *t*-tests and data comparison among multiple groups was done using one-way ANOVA analysis. All statistical analyses were conducted using SPSS version 20.0 software (IBM Corp., Armonk, NY, USA). The *P* value less than 0.05 was considered as statistically significant.

## 3. Results

### 3.1. LQF Alleviates Cardiac Dysfunction of MI Mice

**Figure 1:**
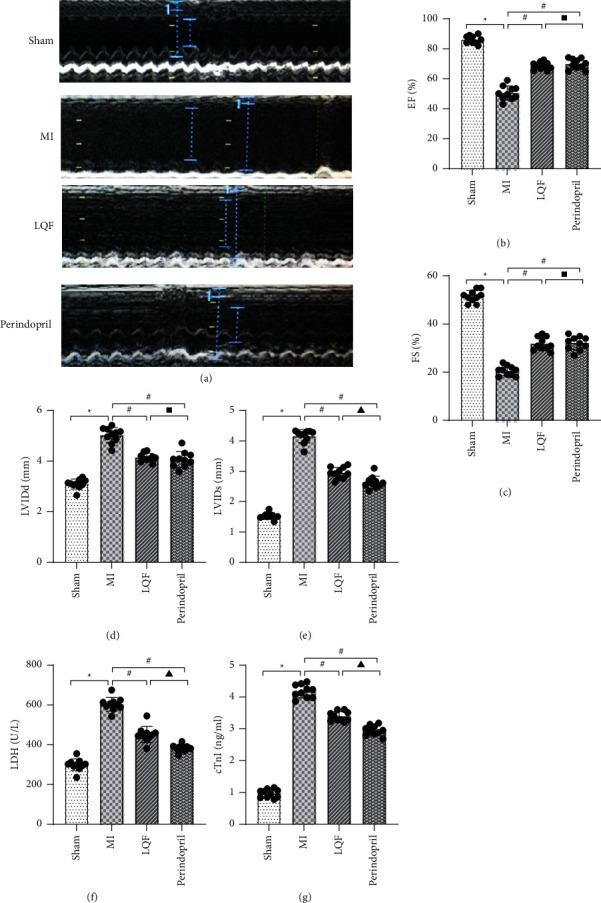
LQF alleviates cardiac dysfunction of MI mice. (a) Representative M-mode images of each group. (b–e) EF, FS, LVEDd, and LVIDs in each group. *n* = 10 per group. ^*∗*^*P* < 0.05 versus sham group. #*P* < 0.05 versus MI group. Δ*P* < 0.05 versus the perindopril group. *P* > 0.05 versus the perindopril group.

Echocardiography is an important method to evaluate the changes of cardiac structure and function. In this study, echocardiography was used to explore the effect of LQF on MI mice. The results showed that, compared with the sham group, EF and FS in the MI group were significantly decreased, while LVEDd and LVIDs were significantly increased, suggesting that the left ventricular wall contraction ability of mice in the MI group was decreased. Compared with MI group, EF and FS in LQF group and perindopril group were significantly increased, while LVEDd and LVIDs were significantly decreased, indicating that LQF and perindopril can improve cardiac function in MI mice (Figures 1(a)–1(g)). Myocardial injury markers are usually used as diagnostic indicators of MI; among them, LDH and cTnI are commonly used to reflect the severity of myocardial injury. The ELISA results showed that (Figures 1(f) and 1(g)), compared with the sham group, the serum levels of LDH and cTnI of MI group mice were significantly increased. Compared with MI group, the levels of LDH and cTnI in LQF group and perindopril group were significantly reduced, indicating that LQF and perindopril can reduce the myocardial injury of MI mice.

### 3.2. LQF Improved Myocardial Injury and Inflammatory Response of MI Mice

**Figure 2:**
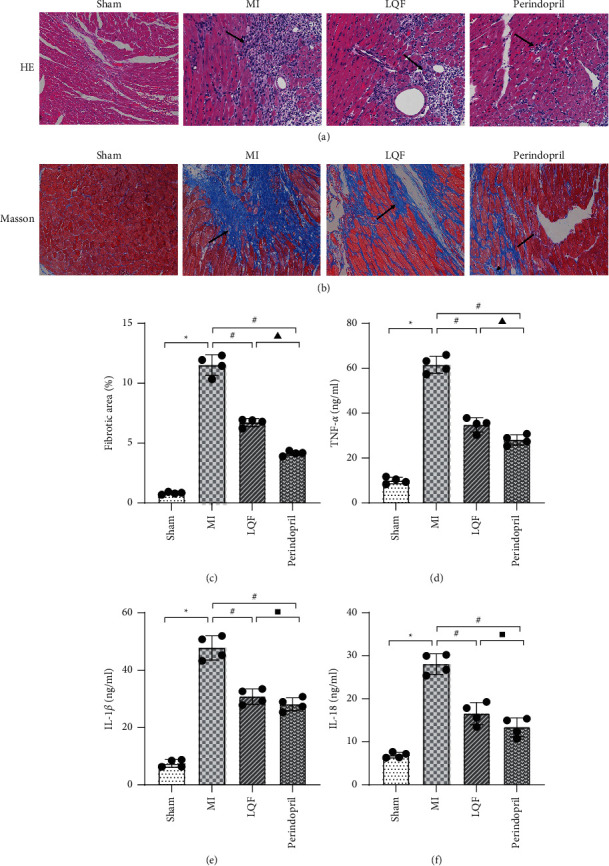
LQF improved myocardial injury and inflammatory response of MI mice. (a) Representative H&E staining. Scale bar = 200 *μ*m; *n* = 4 per group. (b) Representative Masson staining. Scale bar = 200 *μ*m; *n* = 4 per group. (c) The percentage of fibrosis area in the groups. ((d)–(f)) Serum TNF-*α*, IL-1*β*, and IL-18 levels of mice in each group. *n* = 4 per group. ^*∗*^*P* < 0.05 versus sham group. #*P* < 0.05 versus MI group. Δ*P* < 0.05 versus the perindopril group. *P* > 0.05 versus the perindopril group.

4 weeks after MI, H&E staining showed that the cardiomyocytes morphology in the sham group was normal, and muscle fibers were arranged orderly, while in the MI group, myocardial cells were edema, muscle fibers were arranged disorderly and broken, and intercellular edema and inflammatory infiltration were also observed. Compared with the MI group, LQF group and perindopril group contained significantly fewer swollen cells, and the arrangement of muscle fibers was regular (Figure 2(a)). Masson staining showed that there were only a few collagen fibers in the sham group, and the MI group had a large number of blue collagen fibers distributed around cardiomyocytes, while collagen fibers in LQF group and perindopril group were significantly lower than those in MI group (Figure 2(b)). Inflammatory response plays an important role in MI [14]. Therefore, we detected the effect of LQF on myocardial inflammatory factors in MI mice. ELISA results showed that serum levels of inflammatory cytokines TNF-*α*, IL-1*β*, and IL-18 in MI group were significantly higher than those in sham group. The levels of TNF-*α*, IL-1*β*, and IL-18 in LQF and perindopril groups were significantly less than those in the MI group (Figures 2(d)–2(f)), suggesting that LQF can alleviate myocardial inflammation induced by MI.

### 3.3. LQF Alleviated Myocardial Fibrosis of MI Mice

**Figure 3:**
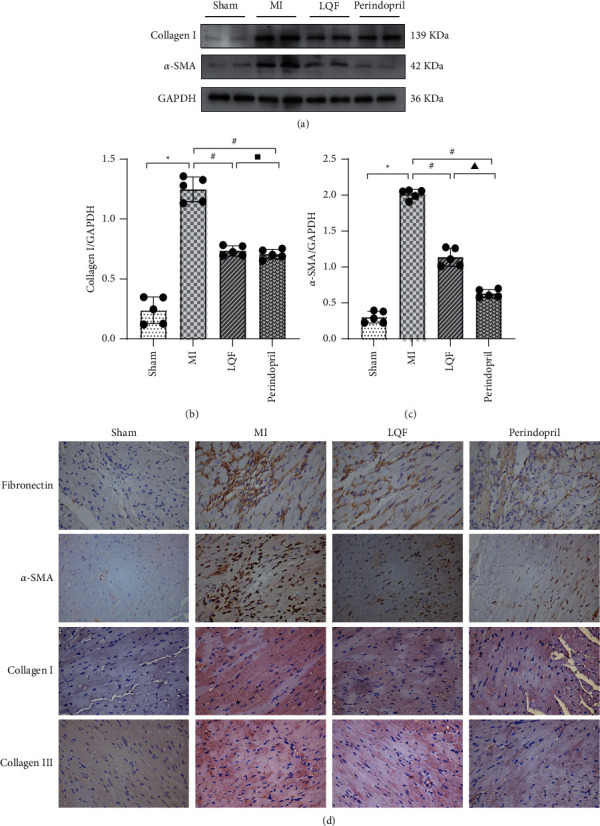
LQF alleviated myocardial fibrosis of MI mice. (a) Representative western blots of collagen I and *α*-SMA (*n* = 5 per group). ((b), (c)) Collagen I and *α*-SMA relative expression levels in each group. ^*∗*^*P* < 0.05 versus the sham group. #*P* < 0.05 versus the MI group. Δ*P* < 0.05 versus the perindopril group. ^▪^Δ*P* > 0.05 versus the perindopril group. (d) Representative images of immunohistochemical localizations of Fibronectin, *α*-SMA, collagen I, and collagen III. Scale bar = 400 *μ*m.

It is well established that inflammation response aggravates myocardial fibrosis; therefore, we detected the expression of fibrotic protein in myocardial tissue. The protein expression levels of collagen I and *α*-SMA in the MI group were significantly upregulated; strikingly, LQF and perindopril markedly reduced the expressions of collagen I and *α*-SMA in myocardium (Figures 3(a)–3(c)). Furthermore, immunohistochemical staining showed that the expressions of Fibronectin, *α*-SMA, collagen I, and collagen III were decreased in the LQF group and the perindopril group, which further confirmed the antifibrosis effect of LQF and the perindopril.

### 3.4. LQF Inhibited the Activation of NLRP3 Inflammasome of MI Mice

**Figure 4:**
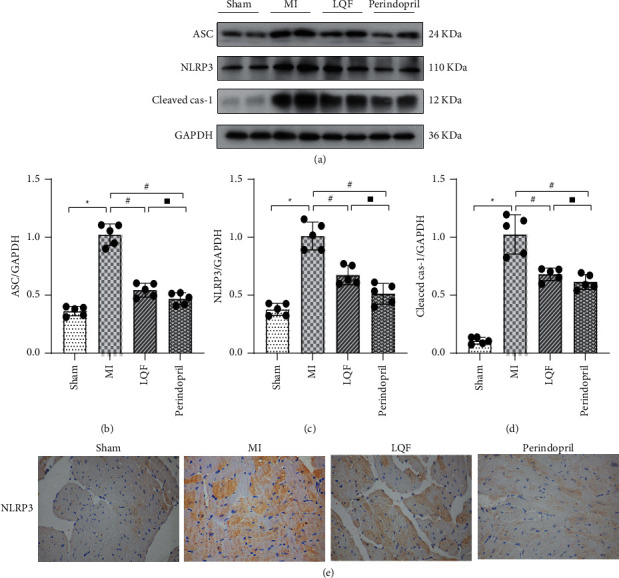
LQF inhibited the activation of NLRP3 inflammasome of MI mice. (a) Representative western blots of NLRP3 (*n* = 5 per group). ((b)–(d)) The relative expression levels of these proteins in each group. ^*∗*^*P* < 0.05 versus the sham group. #*P* < 0.05 versus the MI group. ^▪^Δ*P* > 0.05 versus the perindopril group. (e) Representative images of immunohistochemical localizations of NLRP3. Scale bar = 200 *μ*m.

It is well known that the NLRP3 inflammasome is extensively involved in myocardial fibrosis [15]. Therefore, to clarify whether the effect of LQF in reducing myocardial fibrosis is related to inhibited NLRP3 inflammasome, next, we observed the expression of NLRP3 inflammasome in myocardial tissue of MI mice. As displayed in Figures 4(a)–4(d), the NLRP3, ASC, and cleaved caspase-1 were significantly upregulated in MI group, which indicated that MI promoted the activation of NLRP3 inflammasome. Administration of LQF and perindopril could significantly decrease the expressions of NLRP3, ASC, and cleaved caspase-1 in MI mice. What is more, immunohistochemical analysis also demonstrated that LQF and perindopril could inhibit the expression of NLRP3 after MI (Figure 4(e)). These findings indicate that the effect of LQF on myocardial fibrosis is associated with inhibition of NLRP3 inflammasome.

### 3.5. LQF Inhibits TLR4/MyD88/NF-*κ*B Pathway of MI Mice

**Figure 5:**
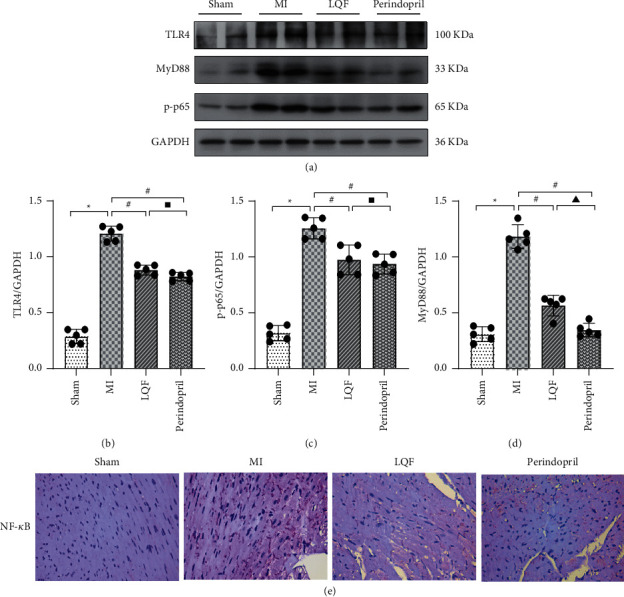
LQF inhibits TLR4/MyD88/NF-*κ*B pathway of MI mice. (a) Representative western blots of TLR4, MyD88, and p-p65 (*n* = 5 per group). ((b)–(d)) The relative expression levels of these proteins in each group. ^*∗*^*P* < 0.05 versus the sham group. #*P* < 0.05 versus the MI group. ^▲^Δ*P* < 0.05 versus the perindopril group. ^▪^Δ*P* > 0.05 versus the perindopril group. (e) Representative images of immunohistochemical localizations of NF-*κ*B. Scale bar = 200 *μ*m.

TLR4/MyD88/NF-*κ*B signaling pathway is one of the important mechanisms to activate NLRP3 inflammasome; to further explore the mechanism by which LQF inhibits NLRP3 inflammasome activation, we examined the effect of LQF on TLR4/Myd88/NF-*κ*B signaling pathway. Our western blot results showed that TLR4, MyD88, and p-p65 were significantly increased in the MI group. Strikingly, the levels of TLR4, MyD88, and p-p65 were markedly reduced in the LQF and perindopril groups. These observations illustrated that LQF and perindopril could via downregulating the TLR4/MyD88/NF-кB signaling pathway inhibit the activation of NLRP3 inflammasome (Figures 5(a)–5(d)). Moreover, immunohistochemical analysis also demonstrated that LQF and perindopril markedly suppressed the NF-*κ*B expression (Figure 5(e)). All these results indicate that LQF inhibited NLRP3 inflammasome activation after MI, at least partly, through inhibiting the TLR4/MyD88/NF-*κ*B signaling pathway.

## 4. Discussion

Here, we verified that LQF could downregulate TLR4/MyD88/NF-*κ*B signaling pathway and inhibit NLRP3 inflammasome activation of MI mice. These changes could reduce the expressions of collagen I and *α*-SMA, thus reducing myocardial fibrosis and improving cardiac function. Together, these findings in the present study indicate that LQF alleviated myocardial fibrosis owing to inhibiting TLR4/MyD88/NF-*κ*B signaling pathway and inhibited NLRP3 inflammasome activation. In addition, we also showed that perindopril has the same mechanism of ameliorate myocardial fibrosis. These findings suggest the potential clinical application of LQF to treat myocardial fibrosis.

Myocardial fibrosis refers to the excessive accumulation of collagen fibers in the normal tissue structure of myocardium and the significant increase of collagen concentration in heart tissue, resulting in the increase of cardiac stiffness and the decrease of cardiac function; myocardial fibrosis is the main pathophysiological process of MI, which can lead to cardiac remodeling and gradually develop into chronic HF [16]. Several mechanisms are involved in the pathogenesis of myocardial fibrosis; among them, inflammatory response plays an important role in the occurrence and development of myocardial fibrosis, which leads to cardiac fibrosis by promoting collagen deposition [17]. Myocardial ischemia induces an inflammatory response, and a moderate inflammatory response facilitates the removal of necrotic cell debris from the infarct area. If there is no appropriate anti-inflammatory treatment, it will aggravate this inflammatory response and induce the release of various proinflammatory factors and profibrotic mediators, such as interleukin-1 (IL-1), tumor necrosis factor-*α* (TNF-*α*), transforming growth factor-*β*1 (TGF-*β*1), and platelet derived growth factor (PDGF). Mei Sun et al. found that TNF-*α* mediates cardiac remodeling and cardiac dysfunction under a pressure overload mice model, while TNF-*α* knockout mice showed decreased inflammatory response and alleviated myocardial fibrosis [18]. Kraft et al. demonstrated that IL-1*β* antibodies can reduce virus induced myocardial injury, inflammation response, and subsequent myocardial fibrosis [19]. Therapeutic strategies for different inflammatory response show that it can effectively reduce MI area and myocardial fibrosis [20]. Therefore, inhibiting inflammatory response has become an important strategy to reduce cardiac injury and improve myocardial fibrosis [21–23]. Consistent with this notion, our study found that EF and FS in LQF group were significantly increased, while LVEDd and LVIDs were significantly decreased. LDH and cTnI, which have high sensitivity to evaluate myocardial injury, both reduced in the LQF group. Moreover, the cytokine levels of TNF-*α*, IL-1*β*, and IL-18 were significantly reduced in LQF group, suggesting that LQF can alleviate myocardial inflammation induced by MI, thereby improving the cardiac function.

NLRP3 inflammasome is a key regulator of immune response. As a sensor, NLRP3 can recognize a variety of danger signals and trigger inflammatory response and pyroptosis. In the past decade, NLRP3 inflammasome has been demonstrated to be widely involved in myocardial fibrosis [15, 24, 25]. The underlying mechanism may be related to regulation with pyroptosis [26], mitochondrial function [27], and myofibroblast differentiation [28]. It is well established that NLRP3 inflammasome is upregulated in the process of MI [29, 30]. MCC950, a speciﬁc NLRP3 inhibitor, can effectively decrease the deposition of collagen I, collagen III, and *α*-SMA [31]. Inhibition of ASC and caspase-1 could decrease inflammatory responses and myocardial fibrosis [32]. Therefore, NLRP3 inflammasome may be a potential target for the treatment of myocardial fibrosis. In the present study, we observed that *α*-SMA, collagen I, collagen III, and Fibronectin, the markers of cardiac fibrosis, were decreased in LQF group, suggesting that treatment with LQF reduced myocardial fibrosis. Moreover, LQF group inhibited the protein expressions of NLRP3, ASC, and cleaved caspase-1; these data supported the notion that LQF alleviates myocardial fibrosis by inhibiting the activation of NLRP3 inflammasome.

TLR4/MyD88/NF-*κ*B signaling pathway is the main regulatory pathway of NLRP3 inflammasome activation [33]. TLR4 is an important pattern recognition receptor in the nonspecific immune system. It is activated by PAMPs or DAMPs in the body and then starts MyD88 dependent signal transduction pathway to activate NF-*κ*B signal path [34]. In the resting state, NF-*κ*B binds to inhibitory factor I*κ*B as a dimer and is inactive. When activating the MyD88 dependent signaling pathway, MyD88 activates the I*κ*B kinase through downstream bridging molecules and phosphorylates and degrades I*κ*B, and NF-*κ*B is activated and translocated into the nucleus [35], initiating transcription and expression of NLRP3 and IL-1*β* precursors. Subsequently, PAMPs or DAMPs induce the activation of NLRP3 inflammasome, leading to a series of inflammatory cascades. Therefore, TLR4/MyD88/NF-*κ*B pathway plays a crucial role in NLRP3 inflammasome mediated inflammatory response. Studies have shown that tanshinone IIA can inhibit TLR4/NF-*κ*B/NLRP3 signaling pathway reducing myocardial injury [36]. In addition, intervention with TLR4 specific inhibitor TAK-242 can inhibit NLRP3 inflammasome activation caused by coronary microembolization [37, 38], suggesting that TLR4/MyD88/NF-*κ*B pathway and NLRP3 inflammasome mediate the occurrence of inflammatory response in myocardial injury. In the current study, the results showed that LQF downregulated the TLR4/MyD88/NF-*κ*B signaling pathway. Taken together, these studies strongly implicate that LQF inhibited TLR4/MyD88/NF-*κ*B signaling pathway and NLRP3 inflammasome activation.

In conclusion, LQF may downregulate TLR4/MyD88/NF-*κ*B pathway and inhibit the activation of NLRP3 inflammasome, thereby reducing inflammatory response and myocardial fibrosis. This study deepened the understanding of the anti-inflammatory mechanism and improving myocardial injury of LQF, provided a foundation for further in-depth study of LQF, and also provided new targets and new ideas for the clinical prevention and treatment of MI.

### 4.1. Limitations

In the present study, we just demonstrated that LQF can downregulate TLR4/MyD88/NF-*κ*B pathway and inhibit NLRP3 inflammasome activation in MI mice, but no further cell experiments were carried out.

## Data Availability

The datasets are available from the corresponding author upon reasonable request.
